# Milk Alternatives and Non-Dairy Fermented Products: Trends and Challenges

**DOI:** 10.3390/foods10020222

**Published:** 2021-01-21

**Authors:** Erica Pontonio, Carlo Giuseppe Rizzello

**Affiliations:** 1 Department of Soil, Plant and Food Science, University of Bari Aldo Moro, Giovanni Amendola 165/A, 70126 Bari, Italy; 2 Department of Environmental Biology, “Sapienza” University of Rome, Piazzale Aldo Moro 5, 00185 Rome, Italy; carlogiuseppe.rizzello@uniroma1.it

The growing prevalence of allergenicity towards cow’s milk, lactose intolerance, and hypercholesterolemia, as well as the trend of plant-based diets (vegetarian and vegan), is pushing the food industry and the global market towards the design, supply and production of novel plant-based milk alternatives [1]. Today, milk alternatives are commercially obtained from a variety of plant-derived ingredients, such as cereals, legumes, pseudo-cereals, nuts, and fruits. In particular, plant-based beverages and yogurt-like products obtained with oat, rice, quinoa, soy, almond, coconut, hazelnut, sesame, and hemp, are the most commonly consumed [1,2].

Depending on the raw materials and technology employed in the production processes, large nutritional composition variability, and significant differences from milk counterparts in terms of technological and sensory features have been reported [2,3].

Indeed, although several efforts have been made, the major challenges still faced by producers of plant-based beverages and yogurt-like products are associated with the appearance and texture properties, unbalanced nutritional profile, and the presence of anti-nutritional compounds [1,3,4,5]. Product stability is an important parameter of plant-based beverages and yogurt-like products in which particles can easily aggregate and precipitate resulting in serum separation [1,6]. Innovative food processing technologies display great potential to improve the storage stability of these products and, among all, the combined use of calcium and thermal treatments proved to be successful for the aggregation of lentil proteins in concentrated emulsions [6].

Among nutritional issues, mainly related to the chemical composition of the raw materials used, the presence of anti-nutritional factors (ANF) which negatively affect the sensory profile as well as the bioavailability of macro- and micro-nutrients and protein quality (as compared to animal derived) are still one of the main concerns [1,4]. Several approaches have been proposed to improve the nutritional profile of plant-based milk alternatives, and the use of novel protein ingredients [7,8] as well as the occurrence of the fermentation process (spontaneous or driven by selected strains) seem to be valuable options to overcome these drawbacks [4].

Protein ingredients can be obtained from plant raw materials through different technological processes such as precipitation, fractionation, and extraction. Depending on the plant-matrix and the process employed, techno-functional and nutritional properties greatly change, requiring tailored investigations of the different options [7,8].

Fermentation with ad hoc-selected lactic acid bacteria often results in improved of nutritional profile mainly due to the release of amino acids and bioactive compounds, decrease of ANF by direct (microbial enzymatic activities) and indirect (activation of endogenous enzymes) activities and, enhancement of the protein in vitro digestibility and antioxidant potential [4,9].

The suitability of plant-based milk alternatives as probiotic carrier has been investigated [4,10,11]. The need of an accurate selection of the probiotic strain in order to avoid the negative effects on the microbial survival in the non-dairy substrate, such as the onset of unpleasant sensory characteristics related to fermentation, has been highlighted [4,10,11].

The use of probiotic strains deriving from well-characterized functional foods or beverages, such as kefir [11], moreover selected for the capability to conduct the fermentation of the specific plant-based matrix, might represent a promising approach to guarantee the best adaptation and to prevent undesirable effects [4,10,11].

A further growth is expected in the sector of plant-based milk alternatives in future, thanks to the great consumer interest and the ample opportunity to differentiate final products based on plant ingredients and production technologies.

Concerted research efforts are required in the coming years to provide, besides suitable and affordable bioprocessing options, tailor-made products with specific technological, nutritional, and sensory properties.

## References

[1] Munekata P.E.S., Domínguez R., Budaraju S., Roselló-Soto E., Barba F.J., Mallikarjunan K., Roohinejad S., Lorenzo J.M. (2020). Effect of Innovative Food Processing Technologies on the Physicochemical and Nutritional Properties and Quality of Non-Dairy Plant-Based Beverages. Foods.

[2] Grasso N., Alonso-Miravalles L., O’Mahony J.A. (2020). Composition, Physicochemical and Sensorial Properties of Commercial Plant-Based Yogurts. Foods.

[3] Angelino D., Rosi A., Vici G., Russo M.D., Pellegrini N., Martini D., On behalf of the SINU Young Working Group (2020). Nutritional Quality of Plant-Based Drinks Sold in Italy: The Food Labelling of Italian Products (FLIP) Study. Foods.

[4] Verni M., Demarinis C., Rizzello C.G., Baruzzi F. (2020). Design and Characterization of a Novel Fermented Beverage from Lentil Grains. Foods.

[5] Bonke A., Sieuwerts S., Petersen I.L. (2020). Amino Acid Composition of Novel Plant Drinks from Oat, Lentil and Pea. Foods.

[6] Alonso-Miravalles L., Zannini E., Bez J., Arendt E.K., O’Mahony J.A. (2020). Thermal and Mineral Sensitivity of Oil-in-Water Emulsions Stabilised using Lentil Proteins. Foods.

[7] Vogelsang-O’Dwyer M., Petersen I.L., Joehnke M.S., Sørensen J.C., Bez J., Detzel A., Busch M., Krueger M., O’Mahony J.A., Arendt E.K. (2020). Comparison of Faba Bean Protein Ingredients Produced Using Dry Fractionation and Isoelectric Precipitation: Techno-Functional, Nutritional and Environmental Performance. Foods.

[8] Vogelsang-O’Dwyer M., Bez J., Petersen I.L., Joehnke M.S., Detzel A., Busch M., Krueger M., Ispiryan L., O’Mahony J.A., Arendt E.K. (2020). Techno-Functional, Nutritional and Environmental Performance of Protein Isolates from Blue Lupin and White Lupin. Foods.

[9] Łopusiewicz Ł., Drozłowska E., Siedlecka P., Mężyńska M., Bartkowiak A., Sienkiewicz M., Zielińska-Bliźniewska H., Kwiatkowski P. (2019). Development, Characterization, and Bioactivity of Non-Dairy Kefir-Like Fermented Beverage Based on Flaxseed Oil Cake. Foods.

[10] Bancalari E., Castellone V., Bottari B., Gatti M. (2020). Wild *Lactobacillus casei* Group Strains: Potentiality to Ferment Plant Derived Juices. Foods.

[11] de Oliveira Coelho B., Fiorda-Mello F., de Melo Pereira G.V., Thomaz-Soccol V., Rakshit S.K., de Carvalho J.C., Soccol C.R. (2019). In Vitro Probiotic Properties and DNA Protection Activity of Yeast and Lactic Acid Bacteria Isolated from A Honey-Based Kefir Beverage. Foods.

